# Case management-integrated supported employment for individuals with schizophrenia: a 3-year longitudinal study in China

**DOI:** 10.3389/fpsyt.2026.2001172

**Published:** 2026-10-30

**Authors:** Jiangchan Li, Kan Yang, Longrun Guo, Dongsheng Zhou, Dan Huang

**Affiliations:** 1 The Third People’s Hospital of Ganzhou City, Ganzhou, China; 2 Ganzhou Key Laboratory of Physical Diagnosis, Treatment and Rehabilitation for Mental Disorders, Ganzhou, China; 3 The Affiliated Kangning Hospital of Ningbo University, Ningbo, China

**Keywords:** case management, longitudinal study, psychiatric rehabilitation, schizophrenia, supported employment

## Abstract

**Background:**

Although case management (CM) and supported employment (SE) have each demonstrated effectiveness for individuals with schizophrenia, evidence on their long-term combined effects remains limited, particularly in real-world settings.

**Methods:**

This 3-year longitudinal randomized study enrolled 60 patients with schizophrenia. Participants were randomly assigned in a 1:1 ratio to a CM-integrated SE (CM-SE) group (n = 30) or an independent employment (IE) group (n = 30). Employment stability, psychiatric symptoms (PANSS), and perceived social support (PSSS) were assessed at baseline, 12, 24, and 36 months.

**Results:**

The CM-SE group demonstrated significantly longer cumulative employment duration (median 25.0 vs 15.0 months, P<0.001), greater improvements in PANSS scores (time×group interaction, P<0.001), and progressively enhanced PSSS scores (time×group interaction, P<0.001) compared to the IE group.

**Conclusions:**

The CM-SE model significantly improves long-term employment stability, psychiatric symptoms, and social support in individuals with schizophrenia, supporting its implementation as an integrated psychosocial rehabilitation strategy.

## 1 Introduction

Schizophrenia is a severe and chronic mental disorder that imposes a substantial burden on individuals, families, and healthcare systems globally (1, 2). Characterised by persistent positive and negative symptoms, cognitive deficits, and profound disruptions in social and occupational functioning, the illness often follows a relapsing course, leading to progressive functional decline (3). Despite advancements in pharmacotherapy, significant gaps remain in achieving full functional recovery (4). High relapse rates, poor treatment adherence, and severe social and vocational impairments are pervasive challenges, underscoring the urgent need for effective psychosocial interventions (5, 6).

Case management (CM) has emerged as a cornerstone of community-based care for individuals with severe mental illness (SMI) (6, 7). Originating as a mechanism to coordinate fragmented services, CM has evolved into comprehensive models that provide long-term, individualised support (8). Meta-analyses have demonstrated that CM can improve clinical outcomes, enhance social functioning, and reduce hospitalisation rates (9). For instance, a recent real-world pilot study by Zhang et al. (2025) found that while CM did not significantly reduce relapse rates over a six-month period, it was associated with significant improvements in self-efficacy and social functioning, highlighting its value in enhancing personal recovery (10). Similarly, a 24-month study by Incidere and Yildiz (2019) in Turkey demonstrated that a hybrid case management model led to significant reductions in hospitalisations and improvements in clinical and social functioning, particularly when combined with psychosocial skills training and family involvement (11). Crucially, however, the core of CM is service coordination and continuity of care rather than vocational outcomes: reviews of assertive community treatment and intensive case management found little effect on vocational functioning (12). CM alone therefore does not directly improve competitive employment, even when clinical and social domains do improve.

Previous literature indicates that the employment rate of patients with schizophrenia is significantly lower than that of the general population (13), contributing to social marginalisation, poverty, and poor quality of life (14). Although traditional vocational rehabilitation has shown limited success, supported employment (SE), particularly the Individual Placement and Support (IPS) model, has demonstrated robust evidence in helping individuals with SMI obtain and maintain competitive employment by integrating vocational and mental health services and focusing on rapid job placement (15, 16). Systematic reviews have confirmed that SE is superior to traditional vocational rehabilitation in achieving competitive employment outcomes (17–19). At the same time, SE’s effectiveness depends on clinical stability, treatment adherence, and sustained non-vocational support — precisely the functions that SE itself is not designed to provide.

CM and SE are therefore complementary rather than interchangeable. CM supplies the continuous, individualised, strength-based support that keeps participants engaged in care and clinically stable, whereas SE supplies the rapid, competitive-employment focus that CM lacks. Integrating the two should be mutually reinforcing: CM sustains the clinical and social conditions under which an SE placement can succeed, while SE gives CM an explicit functional-recovery goal. Yet the combined effect of these two approaches in schizophrenia remains underexplored.

Thus, we conducted a 3-year longitudinal study to evaluate the efficacy of a novel Case Management-Integrated Supported Employment (CM-SE) model. This model combines the continuous, individualised, and strength-based support of CM with the core principles of SE, including rapid job search and ongoing workplace support. We hypothesised that, compared to independent employment (IE) following standard care, the CM-SE model would lead to (1) longer cumulative employment duration, (2) greater and sustained improvement in psychiatric symptoms, and (3) enhanced perceived social support over a 3-year period.

## 2 Methods

### 2.1 Participants

The study was conducted from October 2021 to September 2024 at the Department of Rehabilitation Medicine, the Third People’s Hospital of Ganzhou, Jiangxi Province, China. The study protocol was approved by the Ethics Committee of the Third People’s Hospital of Ganzhou (Approval No. Gzsyy2021121), and all procedures were performed in full compliance with the 2013 revision of the Declaration of Helsinki. Written informed consent was obtained from all participants and their legal guardians prior to enrollment. The inclusion criteria included: (1) 18–55 years of age; (2) schizophrenia diagnosis confirmed by two independent psychiatrists, based on the International Classification of Mental Disorders, 10th Edition (ICD-10); (3) the included patients had a PANSS total score ≤70 and a positive score ≤18; (4) At least 3 months have passed since the last hospitalization; (5) A stable guardian, i.e., at least one family member who meets the guardian criteria (guardian criteria: one of the parents, spouse, children, or siblings has a junior high school education or higher and is capable of undertaking the tasks of family education and vocational rehabilitation guidance); (6) The patient had no serious somatic disease; (7) Have basic working ability. The exclusion criteria included: (1) Comorbid with substance use disorder, moderate to severe intellectual disability, or other central nervous system diseases that affect cognitive and occupational function; (2) Had participated in other standardized vocational rehabilitation programs within 6 months prior to enrollment; (3) Were unable to complete the 3-year follow-up due to planned migration or other reasons; (4) Had a history of violent or self-injurious behavior within 1 year prior to enrollment that posed a safety risk in the workplace.

### 2.2 Grouping and matching

A total of 60 participants were enrolled in this study, with a 1:1 ratio assigned to the intervention group (CM-SE group, n=30) and the control group (independent employment group, n=30). Participants were randomly assigned in a 1:1 ratio to the CM-SE group (n = 30) or the independent employment (IE) group (n = 30). The allocation sequence was generated using a random number table by a researcher who was not involved in recruitment, and allocation was concealed using sequentially numbered, sealed, opaque envelopes. Outcome assessments were conducted by raters blinded to group allocation. There were no statistically significant differences in all baseline demographic and clinical characteristics between the two groups (all P > 0.05), indicating good comparability.

### 2.3 Sample size calculation

Sample size was calculated using G*Power 3.1.9.7 software for repeated-measures analysis of variance (ANOVA). The following parameters were set: effect size f = 0.25 (medium effect size based on previous related studies), type I error rate α = 0.05 (two-sided), test power (1-β) = 0.8, number of groups = 2, number of repeated measurements = 4, and correlation between repeated measurements = 0.5. The calculation showed that a minimum of 24 participants per group was required. Considering a 20% loss to follow-up rate during the 3-year follow-up, the final sample size was set at 30 participants per group, with a total of 60 participants.

### 2.4 CM-SE group

The core principle of the CM-SE model is ongoing, dynamic, and individualized long-term support throughout the entire employment process, rather than one-time job placement. The intervention was delivered by a multidisciplinary team (MDT) consisting of attending psychiatrists, psychotherapists, intermediate social workers, occupational therapists, and rehabilitation nurses, all of whom received unified standardized training on case management and supported employment before the study. The full intervention process included 4 sequential stages, with core case management services running through the entire cycle. (see **Supplementary Material**).

### 2.5 Independent employment group

Participants in the control group received routine psychiatric discharge follow-up services, including a once-every-3-month telephone follow-up by a community psychiatrist, symptom assessment, medication guidance, and regular outpatient review reminders. No standardized case management services, vocational rehabilitation training, or supported employment support were provided during the follow-up period. Participants sought and maintained employment independently in the competitive labor market, and their employment status was recorded during regular follow-ups. No intervention contamination occurred between the two groups during the study period.

### 2.6 Measures

Data were collected at 4 fixed time points: T0 (baseline, pre-enrollment), T1 (12 months after enrollment), T2 (24 months after enrollment), and T3 (36 months after enrollment, end of follow-up). All outcome assessments were conducted by 2 independent psychiatrists who received unified training and were blinded to the group assignment of participants, to minimize assessment bias.

Positive and Negative Syndrome Scale (PANSS) assesses symptomatology (20). The PANSS is an interview and observation-based scale consisting of 30 items: 7 for the positive scale (PANSS-P), 7 for the negative scale (PANSS-N) and 16 for the general psychopathology scale (PANSS-PG). Each item is evaluated according to a Likert scale of 7 degrees of intensity, where 1 is always equivalent to the absence of symptoms and 7 represents extreme severity. Each PANSS item score was assessed by two psychiatrists, and the average of these scores was calculated to obtain the final score for the current analysis. In this study, the interrater reliability of the PANSS total score, as measured by the correlation coefficient, was above 0.8.

The Perceived Social Support Scale (PSSS) was used to assess the perceptions and feelings of social support. The PSSS comprises three dimensions—family support, friend support, and Other Support (i.e., support from significant others)—with a total of 12 items. The total score ranges from 12 to 84, where higher scores indicate greater individual social support (21, 22).

Employment outcomes. Employment months were defined as the total number of months of paid employment during the 36-month follow-up. Both full-time and part-time employment were included; temporary employment was also included if it was paid. Employment interruptions were not subtracted; overlapping employment periods were not double-counted. Employment information was collected from multiple sources: monthly follow-up records, payroll records, social security records, and employer confirmation. Secondary employment indicators included maximum on-duty time and minimum on-duty time.

### 2.7 Statistical analysis

Statistical analysis was performed using SPSS 23.0 software. Measurement data were expressed as mean ± standard deviation (x̄ ± s), and categorical data as percentages (%). Non-parametric tests were conducted for employment-related indicators because these variables were not normally distributed, as confirmed by Shapiro-Wilk tests. The baseline and follow-up data were used to compare the quantitative variables of the study, employing repeated measures analysis of variance (RM-ANOVA). Mauchly’s test was used to assess sphericity; when sphericity was violated, the Greenhouse-Geisser correction was applied. *Post-hoc* comparisons were adjusted using Bonferroni correction. A p value of less than 0.05, derived from two-tailed tests, was considered to indicate statistical significance.

## 3 Results

### 3.1 Demographics of participants

There were no statistically significant differences between the *CM-SE* group and *IE* group in all baseline indicators, including age (35.10 ± 8.00 years vs 31.53 ± 7.86 years; F = 3.034, df=1, P = 0.087), disease duration (9.43 ± 3.92 years vs 8.93 ± 3.57 years; F = 0.267, df=1, P = 0.607), gender distribution (χ²=0.067, df=1, P = 0.796), marital status (χ²=3.429, df=2, P = 0.180), and educational level (χ²=0.656, df=3, P = 0.883). In terms of clinical baseline indicators, no significant between-group difference was observed in the Positive and Negative Syndrome Scale (PANSS) total score (67.73 ± 6.87 vs 65.93 ± 6.65; F = 1.061, df=1, P = 0.307) and the Perceived Social Support Scale (PSSS) total score (30.83 ± 4.32 vs 30.63 ± 5.49; F = 0.025, df=1, P = 0.876). The above results confirmed that the two groups had good baseline comparability. Further demographic details can be found in **Table 1**.

**Table 1:** Socio-demographic and clinical characteristics in patients with Schizophrenia.

Characteristics	CM-SE (n=30)	IE (n=30)	X^2^/*F*	df	P value
Age,year	35.10±8.00	31.53±7.86	3.034	1	0.087
Disease duration (year)	9.43±3.92	8.93±3.57	0.267	1	0.607
Gender Male female	15 (50.0%) 15 (50.0%)	14 (46.7%) 16 (53.3%)	0.067	1	0.796
Marital status Single/Unmarried Married widowed	18 (60.0%) 10 (33.3%) 2 (6.7%)	24 (80.0%) 4 (13.3%) 2 (6.7%)	3.429	2	0.180
Educational level Primary Junior secondary Senior secondary Tertiary	6 (20.0%) 14 (46.7%) 8 (26.7%) 2 (6.7%)	8 (26.7%) 13 (43.3%) 8 (26.7%) 1 (3.3%)	0.656	3	0.883
PANSS baseline	67.73±6.87	65.93±6.65	1.061	1	0.307
PSSS baseline	30.83±4.32	30.63±5.49	0.025	1	0.876

Data are mean ± SD or n (%). Independent samples t-test or chi-square test was used. CM-SE, case management-integrated assisted employment; IE, independent employment; PANSS, Positive and Negative Syndrome Scale; PSSS, Perceived Social Support Scale.

### 3.2 Employment indicators, relapse, and hospitalization status

The cumulative employment months over the 3-year follow-up, were compared as the primary indicator of long-term employment stability between the two groups using the Mann-Whitney U test (**Table 2**). The median cumulative employment months of the *CM-SE* group was 25.0 months (interquartile range [IQR]: 23.0-26.0), which was significantly higher than that of the *IE* group (median 15.0 months, IQR: 12.0-18.8; Z=-5.921, P<0.001). For relapse and hospitalization outcomes, the CM-SE group had a significantly lower recurrence frequency than the IE group (median 1.0, IQR 1.0-2.0 vs. median 2.0, IQR 1.0-3.0; Z = -3.107, P = 0.022). The number of hospitalizations was also significantly lower in the CM-SE group than in the IE group (median 1.0, IQR 0.0-1.0 vs. median 2.0, IQR 1.0-2.3; Z = -5.581, P < 0.001).

**Table 2:** Employment indicators, relapse, and hospitalization status in both groups.

Variables	CM-SE	IE	Z	P value
Maximum on-duty time (month)	16.0 (13.8,19.0)	13.0 (12.0,14.0)	-3.737	<0.001
Minimum on-duty time (month)	7.0 (6.0,8.3)	4.0 (3.0,5.0)	-4.614	<0.001
employment months	25.0 (23.0,26.0)	15.0 (12.0,18.8)	-5.921	<0.001
recurrence frequency	1.0 (1.0,2.0)	2.0 (1.0,3.0)	-3.107	0.022
number of hospitalizations	1.0 (0.0,1.0)	2.0 (1.0,2.3)	-5.581	<0.001

Data are median (IQR). Mann-Whitney U test was used for between-group comparisons.

CM-SE, case management-integrated assisted employment; IE, independent employment.

### 3.3 Longitudinal changes in psychiatric symptoms

Repeated-measures ANOVA was performed to analyze the changes in PANSS total scores over the 3-year follow-up period between the two groups, and the results are shown in **Table 3**. A significant time×group interaction effect was observed for PANSS total score (F = 24.594, df=3, P<0.001).

**Table 3:** Comparison of PANSS and PSSS among SE and IE groups.

Variables	Follow-up assessment at first year		Follow-up assessment at second year		Follow-up assessment at third year		Repeated measured ANOVA	
	*CM-SE*	*IE*	*CM-SE*	*IE*	*CM-SE*	*IE*	Group x time	
	*M*	*S.D.*	*M*	*S.D.*	*M*	*S.D.*	*F*	*P*
PANSS	65.17±6.47	62.10±11.20	55.97±7.37	66.60±8.76	46.97±7.36	64.33±10.63	24.594	<0.001
PSSS	34.33±7.48	29.40±7.04	38.80±7.59	29.33±8.58	45.33±10.01	31.43±8.97	11.881	<0.001

CM-SE, case management-integrated assisted employment; IE, independent employment; PANSS, Positive and Negative Syndrome Scale; PSSS, Perceived Social Support Scale.

*Post-hoc* analysis revealed that the PANSS total score of the *CM-SE* group decreased continuously with the extension of follow-up time, from 65.17 ± 6.47 at the 1-year follow-up to 55.97 ± 7.37 at the 2-year follow-up, and further to 46.97 ± 7.36 at the 3-year follow-up. In contrast, the *IE* group showed no sustained improvement in psychiatric symptoms, with PANSS total scores of 62.10 ± 11.20, 66.60 ± 8.76, and 64.33 ± 10.63 at the 1-year, 2-year, and 3-year follow-ups, respectively. The *CM-SE* group had a significantly greater improvement in PANSS total score than the *IE* group over the 3-year follow-up period.

### 3.4 Longitudinal changes in perceived social support

As shown in **Table 3**, repeated-measures ANOVA revealed a significant time×group interaction effect for PSSS total score (F = 11.881, df=3, P<0.001). The PSSS total score of the *CM-SE* group increased continuously during the follow-up period, from 34.33 ± 7.48 at the 1-year follow-up to 38.80 ± 7.59 at the 2-year follow-up, and to 45.33 ± 10.01 at the 3-year follow-up. Meanwhile, the PSSS total score of the *IE* group remained stable without significant improvement, with scores of 29.40 ± 7.04, 29.33 ± 8.58, and 31.43 ± 8.97 at the three follow-up time points. The *CM-SE* group had a significantly greater increase in perceived social support level than the *IE* group over the 3-year follow-up.

## 4 Discussion

This 3-year longitudinal quasi-experimental study is among the first to evaluate the long-term efficacy of the CM-SE model in individuals with schizophrenia in China. Our findings demonstrate that, compared to independent employment (IE) following standard care, the CM-SE model significantly improved long-term employment stability, sustained reductions in psychiatric symptoms, and progressively enhanced perceived social support over a 36-month follow-up period. These results provide robust evidence for the synergistic effects of combining continuous case management with evidence-based supported employment in a real-world community setting.

### 4.1 Superiority of the CM-SE model in vocational outcomes

The most striking finding of this study is the significantly longer cumulative employment duration in the CM-SE group compared to the IE group, with a median of 25.0 months versus 15.0 months over the three-year period. Our data suggest that the benefits of an integrated model are not only immediate but also sustainable over the long term, consistent with findings from longer-term SE studies (23). A randomized controlled trial conducted by Knapp et al. in six European cities demonstrated that the Individualized Placement and Support (IPS) model for patients with severe mental disorders significantly outperformed traditional vocational services in facilitating competitive employment acquisition, with superior cost-effectiveness (24).

The success of the CM-SE model can be attributed to its structured, multi-stage process that provides continuous, individualised support, aligns with the principles of the Individual Placement and Support (IPS) model (24). Unlike the “train-and-place” approach used in some traditional vocational programs, our model incorporated rapid job search, job matching based on patient strengths, and crucially, ongoing workplace support from a multidisciplinary team (25). Furthermore, the CM-SE model’s dynamic nature allowed for proactive intervention during periods of symptom exacerbation or workplace stress, preventing job loss and promoting stability (26, 27). This level of support is rarely available in standard care or in fragmented vocational services, highlighting the unique value of a fully integrated CM-SE model.

### 4.2 Impact on psychiatric symptoms and perceived social support

The sustained improvement in PANSS scores in the CM-SE group over the 3-year follow-up contrasts with the stable but unimproved symptom profile in the control group. This finding suggests that meaningful vocational activity, supported by a comprehensive case management system, may contribute to clinical stability and recovery. A systematic review of six RCTs examining IPS, employment, and recovery outcomes found that receipt of the IPS intervention alone did not improve symptom outcomes; however, participants who worked during the study period, whether or not they received IPS, experienced significantly greater improvement in negative symptoms compared with those who did not work (28). Similarly, an international 18-month study of IPS across six European centres reported that those who worked had better global functioning, fewer symptoms, and less social disability at final follow-up, and that greater job tenure was associated with better functioning (29). These findings suggest that the clinical benefits observed in our study may be attributed not to the vocational intervention per se, but to the actual experience of working—an interpretation consistent with the view that employment provides structure, purpose, and social integration, which in turn may support symptom stability. In our model, this potential synergy arises because employment offers these very elements, while case management ensures treatment adherence and psychosocial support. Such a synergistic effect may create a positive feedback loop, wherein vocational engagement supports clinical stability, and ongoing case management reinforces treatment adherence and social integration.

The progressive increase in perceived social support (PSSS scores) in the CM-SE group is another critical finding. Employment served as a natural platform for social integration, enabling participants to build relationships with coworkers, supervisors, and customers (30). This finding supports the social functioning improvements observed in previous vocational rehabilitation studies (31). The CM-SE model further reinforced this by facilitating family involvement and maintaining regular contact with the multidisciplinary team, thereby strengthening the patient’s broader social network. In contrast, the control group’s social support remained stagnant, suggesting that without structured support, individuals with schizophrenia may struggle to expand their social networks independently. This highlights a key mechanism through which CM-SE operates: not only by providing a job, but by rebuilding the social scaffolding necessary for sustained community integration.

This study has several limitations. First, this was a single-center study with a relatively modest sample size (n=60), which may limit the generalisability of our findings to other settings or populations. Future multi-center trials with larger, more diverse cohorts are warranted. Second, while our 3-year follow-up is a major strength, the long-term sustainability of employment and clinical improvements beyond this period remains unknown. Longer-term follow-up studies would be valuable. Third, the study was conducted in a specific urban setting in China, and the applicability of the findings to rural areas or other healthcare systems may be limited due to differences in social welfare systems, labour markets, and cultural attitudes toward mental illness.

In summary, this 3-year longitudinal study demonstrates that a Case Management-Integrated Supported Employment (CM-SE) model is a highly effective intervention for individuals with schizophrenia. Compared to standard care followed by independent employment, the CM-SE model significantly improved long-term employment stability, led to sustained reductions in psychiatric symptoms, and progressively enhanced perceived social support. These findings underscore the critical importance of integrating vocational support into long-term case management to achieve meaningful and lasting functional and clinical recovery. Our results advocate for a paradigm shift in mental health services, moving from a focus on symptom management to a holistic approach that prioritises real-world outcomes like employment and social integration. Further research, including multi-center RCTs and cost-effectiveness analyses, is needed to solidify these findings and facilitate the widespread implementation of this promising model.

## Data Availability

The raw data supporting the conclusions of this article will be made available by the authors, without undue reservation.
