# Supplementary material for: Case management-integrated supported employment for individuals with schizophrenia: a 3-year longitudinal study in China
Source: Front Psychiatry. 2026 Oct 30;17:2001172. doi: 10.3389/fpsyt.2026.2001172 (PMC13759744; doi:10.3389/fpsyt.2026.2001172)
Supplement: Supplementary file 1 [file SupplementaryFile1.docx]

*1.1 Intervention Group: Case Management-Integrated Supported Employment (CM-SE) Program*

The core principle of the CM-SE model is ongoing, dynamic, and individualized long-term support throughout the entire employment process, rather than one-time job placement. The intervention was delivered by a multidisciplinary team (MDT) consisting of attending psychiatrists, psychotherapists, intermediate social workers, occupational therapists, and rehabilitation nurses, all of whom received unified standardized training on case management and supported employment before the study. The full intervention process included 4 sequential stages, with core case management services running through the entire cycle.

*1.1.1 Baseline Assessment and Individualized Plan Development (Pre-employment, T0)*

For each enrolled participant, a dedicated case manager was assigned to establish a one-to-one electronic case file. A comprehensive baseline assessment was conducted, including demographic characteristics, clinical symptoms, occupational interests, labor capacity, social function, and family support system. Based on the assessment results, the MDT team jointly developed an individualized supported employment plan, including adaptive training goals, vocational skill training content, symptom monitoring frequency, and family intervention strategies.

*1.1.2 Adaptive Training (Weeks 1-4)*

This stage focused on helping participants adapt to the workplace environment and work routine. The core content included: familiarization with the workplace, colleagues, and work rules; learning simple repetitive tasks (e.g., document sorting, environmental cleaning, item sorting); establishing a standardized daily work schedule (e.g., on-time attendance, break arrangement). The case manager provided one-to-one on-site guidance, decomposed complex tasks into step-by-step operations, and gave daily positive feedback to reinforce adaptive behaviors.

*1.1.3 Vocational Skill Upgrading Training (Weeks 4-12)*

This stage focused on improving participants' vocational skills, teamwork ability, and problem-solving ability in the workplace. The core content included: targeted training on job-specific skills, teamwork practice, basic emergency response and help-seeking skills, and workplace communication skills. A weekly group supervision meeting was held, where participants shared work difficulties and coping strategies, and the MDT team provided targeted guidance.

*1.1.4 Ongoing On-the-Job Support (Week 12 to Month 36)*

This stage focused on maintaining employment stability, preventing disease relapse, and promoting long-term social integration. The core case management services included:

1. Dynamic symptom monitoring: The case manager conducted a weekly on-site behavioral observation and a monthly PANSS assessment; early warning signs of relapse (e.g., increased positive symptoms, medication non-adherence, sleep disturbance) were identified in a timely manner, and the attending psychiatrist was contacted immediately for intervention.

2. Continuous employment support: The case manager conducted monthly one-to-one vocational guidance, adjusted job content and work intensity according to the participant's symptom changes and functional status, coordinated workplace conflicts, and provided re-employment support for participants who resigned.

3. Family Combined Intervention: The case manager conducted a semi-structured interview with the participant's guardian every 3 months, provided family support skills training, established a two-way feedback mechanism between the family and the MDT team, and mobilized family support resources.

4. Crisis intervention: A standardized emergency termination and intervention process was established for participants with acute symptom exacerbation, including temporary job leave, medication adjustment, psychiatric emergency treatment, and re-adaptation training after symptom stabilization.

*1.1.5 Supported Employment Post Setting*

All posts were set up in the hospital and the community rehabilitation station (Gankang Spiritual Station), with a safe, low-stress, and supportive working environment. The posts included: assistant rehabilitation trainer, safety inspector, supermarket cashier, librarian, handicraft production, cooking training assistant, hospital cleaning, outpatient guide, and community activity room manager. All posts provided paid labor with flexible working hours, and the labor remuneration was paid monthly in accordance with local minimum wage standards.
